# Emerging Trends in Non-Enzymatic Cholesterol Biosensors: Challenges and Advancements

**DOI:** 10.3390/bios12110955

**Published:** 2022-11-01

**Authors:** Mashkoor Ahmad, Amjad Nisar, Hongyu Sun

**Affiliations:** 1 Nanomaterials Research Group, Physics Division, Pakistan Institute of Nuclear Science and Technology (PINSTECH), Islamabad 44000, Pakistan; 2 School of Resources and Materials, Northeastern University at Qinhuangdao, Qinhuangdao 066004, China

**Keywords:** non-enzymatic, electrochemical biosensor, functional nanomaterials, cholesterol, challenges, advancement

## Abstract

The development of a highly sensitive and selective non-enzymatic electrochemical biosensor for precise and accurate determination of multiple disease biomarkers has always been challenging and demanding. The synthesis of novel materials has provided opportunities to fabricate dependable biosensors. In this perspective, we have presented and discussed recent challenges and technological advancements in the development of non-enzymatic cholesterol electrochemical biosensors and recent research trends in the utilization of functional nanomaterials. This review gives an insight into the electrochemically active nanomaterials having potential applications in cholesterol biosensing, including metal/metal oxide, mesoporous metal sulfide, conductive polymers, and carbon materials. Moreover, we have discussed the current strategies for the design of electrode material and key challenges for the construction of an efficient cholesterol biosensor. In addition, we have also described the current issues related to sensitivity and selectivity in cholesterol biosensing.

## 1. Introduction

In recent years, electrochemical biosensing has attracted much attention not only in biomedical but also in environmental monitoring, agriculture, and the food industry [1,2]. The development of an efficient, sensitive, and low-cost electrochemical device that can measure the target analyte with high accuracy is in high demand in the healthcare industry. Human health depends on many complex processes that uninterruptedly take place in the body [3,4]. These phenomena are dependent on the balance of different physiological, biological, and chemical species such as glucose, creatinine, cholesterol and H_2_O_2_ etc. [5,6] Among them, cholesterol is a highly important species, which regulates the processes related to the effective functioning of the heart and related organs. In human blood it is found in two forms: 70% in lipoprotein ester form and 30% as free molecules [7]. The normal blood cholesterol level in a healthy human should be less than 5.17 mM (200 mgdL^−1^), while more than 6.21 mM (240 mgdL^−1^) is regarded as high level, which can result different diseases, e.g., nephrosis, arteriosclerosis, hypertension, brain thrombosis, etc. [8,9]. Therefore, the accurate detection of cholesterol in blood is very important in clinical analysis and diagnosis of diseases.

Up till now, various methods such as fluorometry, fluorescence, potentiometric spectrophotometry, chromatography, colorimetric, electrochemical, and Raman spectroscopy-based platforms have been employed to determine cholesterol [10,11,12]. Compared with other detection methods, the electrochemical technique is preferable due to its low cost, fast recovery time, low detection limit, and being highly sensitive [13,14,15,16]. In this technique, the sensing of cholesterol is performed by two approaches. Firstly, the enzymatic reaction of cholesterol. In this reaction, cholesterol oxidase (ChO_x_) is used to oxidize free-cholesterol tocholest-3-one. During this process, hydrogen peroxide is produced as a by-product. The amount of cholesterol is determined by monitoring the concentration of hydrogen peroxide [17]. However, the enzymatic sensing of cholesterol has some in-built drawbacks, such as high cost and degradation of the enzymes during the storage period, thus limiting the scope of the enzymatic approach. Second is the non-enzymatic approach in which the biocatalytic oxidation of cholesterol occurs. The non-enzymatic devices have attracted much attention due to their higher stability, easy fabrication, reproducibility, cost-effectiveness, and the absence of oxygen constraint [18,19]. However, despite possessing many advantages, non-enzymatic detection of cholesterol presents several challenges, such as the selection of suitable electroactive matrix material, poor selectivity, inadequate sensitivity, detection of the pico-molar concentration of analyte, and low electronic communication between the active sites of materials.

**Figure 1:**
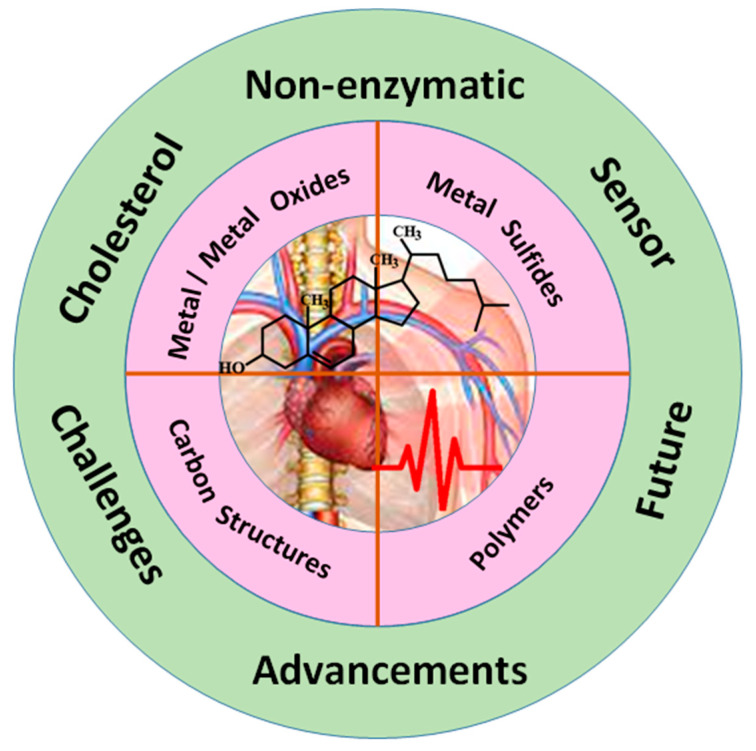
Schematic illustration of nanomaterials-based non-enzymatic cholesterol biosensor.

The scope of this review is to provide an overview of major advances and challenges in the development of an efficient cholesterol biosensor, as illustrated in Figure 1. We have focused on promising recent electroactive nanomaterials, such as metal/metal oxide, metal sulfides, carbon materials, and conductive polymers, which can interact with the cholesterol molecule. We have also described the current strategies for reducing interference to enhance selectivity and sensitivity. Finally, we have summarized the key challenges and issues and future prospects.

## 2. Nanomaterials for Non-Enzymatic Cholesterol Biosensor

In the last few decades, a variety of advanced functional nanomaterials, including zero-dimensional (0D nanoparticles, one-dimensional (1D) nanowires, nanotubes, two-dimensional (2D) transition metal/metal oxide, dichalcogenides, graphenes, and three-dimensional (3D) hierarchical nanoflowers, cubes, and spheres have been developed by employing various synthesis techniques, such chemical vapor deposition, laser ablation, electrospinning, electrodeposition, hydrothermal, vapor phase transport process, sol–gel, and thermal evaporation [20,21,22,23,24,25]. These nanostructures offer unique properties due to controlled morphology, large surface area, and enhanced electrocatalytic properties. In the last decade, these nanostructures have been used in many fields, such as energy storage, optoelectronics, and in the fabrication of non-enzymatic sensors.

### 2.1. Metal/Metal Oxide Cholesterol Biosensor

Since the overall performance of a non-enzymatic biosensor strongly depends on the inherent properties of the electrode materials, in recent years, various metal/metal oxide nanostructures, such as ZnO, TiO_2_, WO_3_, SnO_2_, and Co_3_O_4_, among others, have emerged as promising candidates for the development of efficient and reliable cholesterol biosensors [26,27,28,29,30]. These nanostructure-based electrodes offer various technical advantages, such as a large surface-to-volume ratio, increased active sites, rapid and fast charge transfer rate, and improved electrochemical properties at the nanoscale. Based on their morphological versatility, chemical stability, and their ability to combine in composite structures, they have become highly competitive materials for biosensing [31,32,33]. The composition of the cholesterol sensor is based on the electrochemically active material that is immobilized onto the working electrode using some polymer binder.

Elhag et al. employed the Co_3_O_4_ cotton-like nanostructures for the detection of cholesterol. In this work, they used sodium dodecyl sulfate (SDS) as a template for the synthesis of Co_3_O_4_ nanostructures, which exhibits enhanced sensitivity for cholesterol detection due to its unique morphology [34], ease of preparation, reproducibility, chemical inertness, large surface area, excellent biocompatibility, non-toxicity, chemical and thermal stabilities, fast response, and good reversibility.

**Figure 2:**
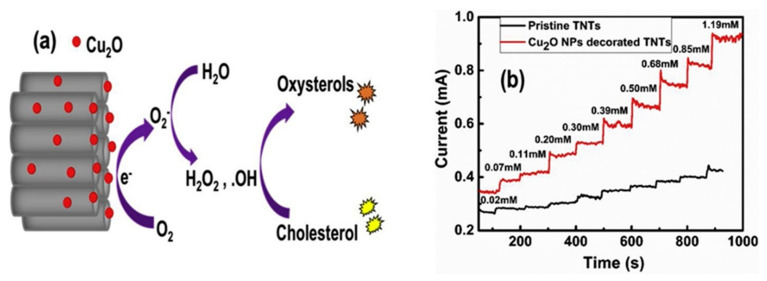
(**a**) Schematic representation of the oxidation of cholesterol at the surface of Cu_2_O NPs-TNTs electrode; (**b**) Amperometry of TNTs and Cu_2_O-decorated TNTs. Reprinted with permission from Ref. [35].

Nanostructured titanium dioxide nanotubes (TNTs) are highly promising materials due to providing a large surface area and biocompatibility. However, their sensing behavior for the measurement of cholesterol analyte has scarcely been investigated. Recently, Khaliq et al. synthesized TiO_2_ nanotubes by anodization of titanium foil and decorated with CuO_2_ nanoparticles using the sonication-assisted chemical bath. A TiO_2_/CuO_2_-based non-enzymatic cholesterol biosensor was fabricated and compared with pristine titanium dioxide nanotubes (TNTs), as shown in Figure 2. The fabricated sensor exhibited an increased oxidation current toward cholesterol detection with much-improved sensitivity and quick response time as compared to the pristine TNTs. Moreover, the sensor also exhibited good thermal stability and demonstrated its practical utility in real physiological conditions [35].

ZnO is an environmentally friendly and biocompatible material that possesses good binding ability with biological entities. Due to these unique features, it has been increasingly employed for the construction of non-enzymatic biosensors for the detection of various analytes, such as glucose, L-cysteine, diclofenac sodium and hydrogen peroxide [36,37,38]. It is the most promising material for the development of highly sensitive electrochemical biosensors due to having versatile properties, such as nontoxicity, biocompatibility, good electrochemical activities, and electron communicating features. Various ZnO nanostructures, such as nanowires, nanoparticles, nanofibers, and flower-shaped ZnO nanostructures have been investigated for the fabrication of enzymatic biosensors [39,40]. Anh et al. developed Ag-ZnO nanorods for the evaluation of their electrochemical behavior for the non-enzymatic detection of cholesterol and compared them with pure ZnO. In this study, it has been found that a Ag-ZnO-NRs-based biosensor showed a linear response in a range of 10–9 mM with improved sensitivity of 135.5 µAmM^−1^cm^−2^ larger than that of ZnO (4.2 µAmM^−1^cm^−2^), and a detection limit of 0.184 mM and 1.78 mM, respectively [41].

One-dimensional nanostructures have attracted wide attention for their potential applications in the field of electronic devices, micro/nanoelectromechanical systems, optoelectronics, field emitters, light-emitting, and electrochemical biosensors providing superior properties. Various types of 1D nanostructures, such as nanowires, nanotubes, and nanorods with controlled structural characteristics are highly significant in the field of electrochemical biosensors. Li et al. synthesized porous tubular Ag nanostructures and investigated their electrochemical properties for non-enzymatic cholesterol detection. In a comparison of solid Ag nanorods, the Ag nanoparticles-modified glassy carbon electrode showed good electrocatalytic activity for cholesterol oxidation in a wide linear range from 2.8 × 10^−4^ M to 3.3 × 10^−2^ M [42].

**Figure 3:**
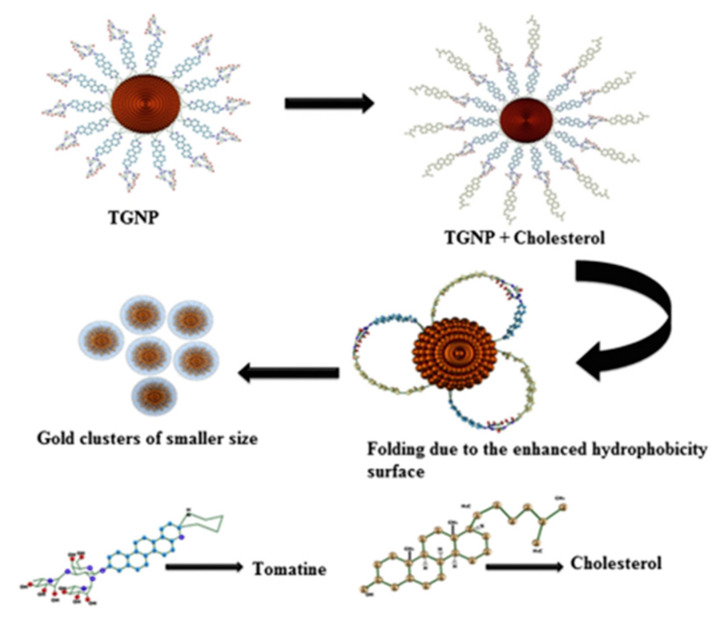
Schematic illustration for the formation of gold nanoparticles by etching of tomatine functionalized gold nanoparticles with cholesterol. Reprinted with permission from Ref. [44].

Yoon et al. reported a cover-type non-enzymatic sensor to determine cholesterol using nanoporous platinum and stainless-steel microneedle patch electrodes. The sensor exhibited enhanced sensitivity of 305 nAmM^−1^ cm^−2^ in 0.1 M phosphate buffer solution [43]. The colloidal gold provides more flexibility during the transformation of the gold nanoparticles’ surface with the change of functional groups. To measure the cholesterol in human serum samples, Raj et al. reported an enzyme-free assay. The developed electrode exhibited a significant linear response for cholesterol within the range of 100,800 ng/mL, with a correlation coefficient (*R*^2^ = 0.9958), and detection limit of 7075 ng/mL. The etching of tomatine functionalized gold nanoparticles in the presence of cholesterol to form small gold nanoparticles is demonstrated by the schematic diagram in Figure 3 [44].

**Figure 4:**
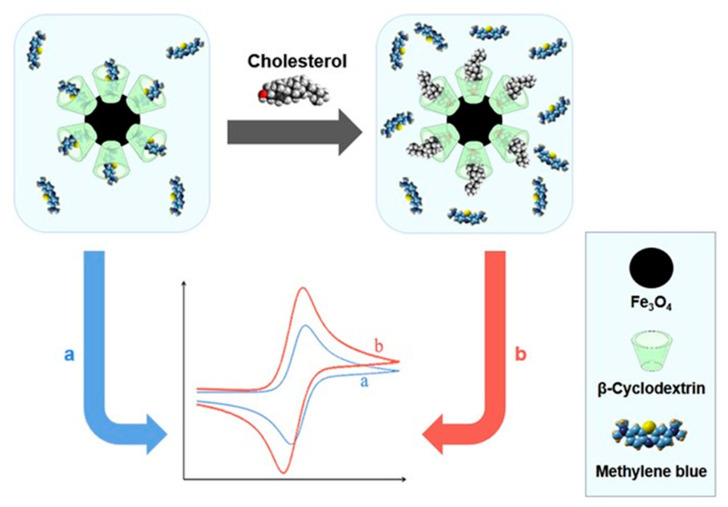
β-Cyclodextrin/Fe_3_O_4_nanocompositefor Cholesterol Sensor. Reprinted with permission from Ref. [47].

Polyoxometalate (POM) nanoclusters showing versatile structures and tunable redox properties can be considered advanced multifunctional structures for sensing applications but these materials are scarcely investigated for the detection of cholesterol. In electrochemical reactions, these materials increased the electron transfer rate, and enhanced electrochemical active surface area and sensitivity [45]. Thakur et al. reported the fabrication of a novel electrochemical biosensor based on poly(ionic liquid)–cobalt polyoxometalate supported on a carbonaceous materials composite. The electrode exhibited an excellent sensitivity of 64 µAµM^−1^cm^−2^ for the detection of cholesterol with the lowest detection limit of (1 × 10^−15^ M). Moreover, the electrode showed two wide linear ranges from 1 fM–200 nM and 0.5 µM–5 mM. The developed sensor also showed a good response for human blood serum samples [46]. Willyam et al. reported a non-enzymatic cholesterol biosensor based on β-cyclodextrin/Fe_3_O_4_ nanocomposite, as illustrated in Figure 4. The developed biosensor reduces the overall analysis time and simplifies the sample measurement procedures, making it suitable for practical applications. Moreover, the sensor showed excellent accuracy and good linearity in the range of 0–150 μM with a detection limit of ~2.88 μM [47].

**Figure 5:**
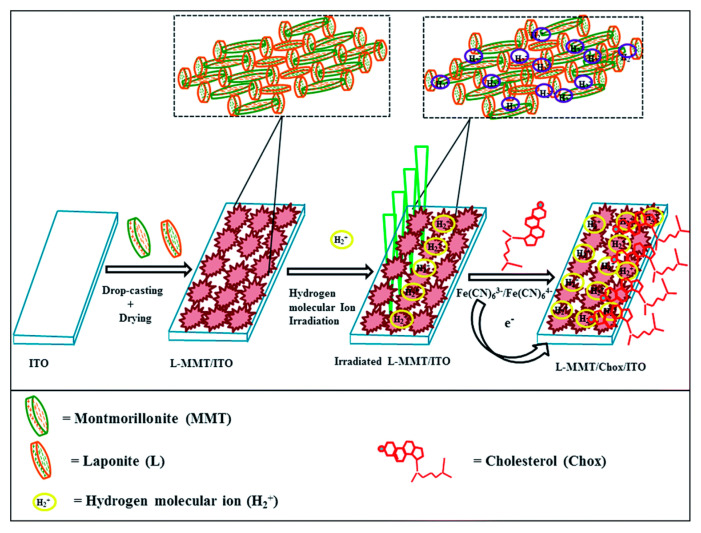
Schematic fabrication and irradiation of L-MMT/ITO film with H^2+^ ion beam for sensing cholesterol. Reprinted with permission from Ref. [48].

Joshi et al. studied the effect of hydrogen ion implantation on the synthetic nanoclay electrode for non-enzymatic sensing of cholesterol. The developed LAPONITE^®^-montmorillonite/indium tin oxide (L-MMT/ITO) film displays about a 20% increase in sensitivity for cholesterol detection at the ion fluence of ~1013 ions per cm^2^ [48]. The performance of the sensor is illustrated by schematic as shown in Figure 5.

### 2.2. Metal Sulfides Biosensor 

**Figure 6:**
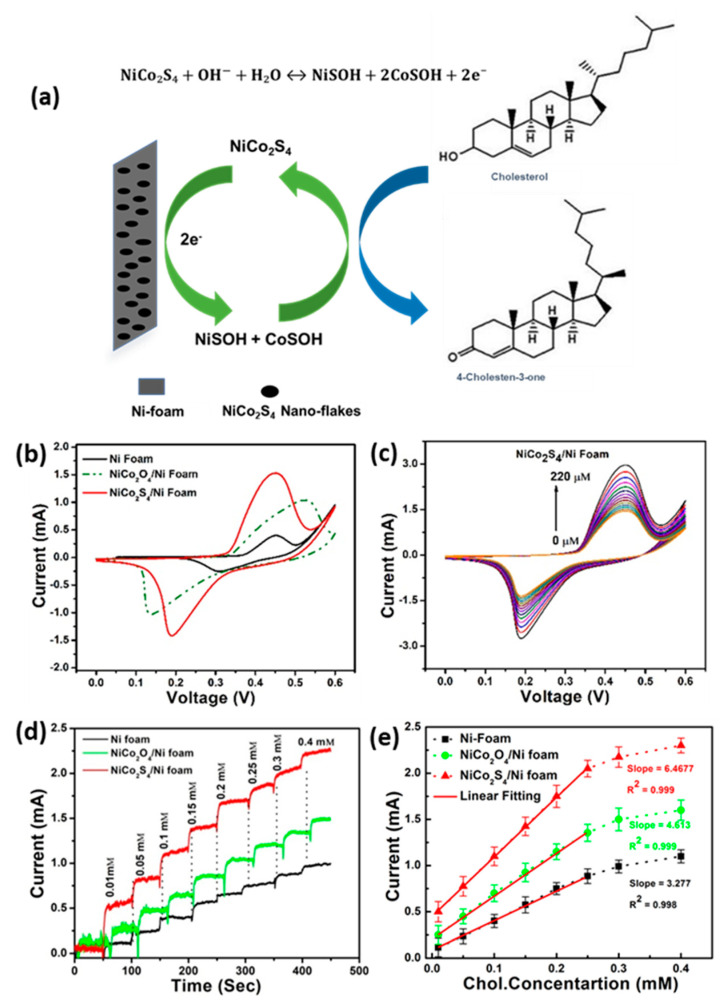
(**a**) Schematic illustration of NiCo_2_S_4_@NF electrode for the measurement of cholesterol. (**b**) Comparative CV response of Ni foam, NiCo_2_O_4_@NF and NiCo_2_S_4_@NF electrodes with the addition of 10 µm cholesterol. (**c**) CV response of NiCo_2_S_4_@Ni-foam electrodes under different concentrations of cholesterol. (**d**) Comparative amperometric response of Ni foam, NiCo_2_O_4_@Ni-foam and NiCo_2_S_4_@Ni-foam electrodes with different concentrations of cholesterol at +0.45 V. (**e**) The calibration plots corresponding to amperometric response. Reprinted with permission from Ref. [58].

Numerous metal sulfides have been investigated for the construction of non-enzymatic biosensors due to their tunable band gaps, diverse crystal structure, rich surface, redox chemical, and the existence of multivalence cations [49,50,51,52]. Among various sulfides, NiCo_2_S_4_ is a promising electrode material for the fabrication of cholesterol biosensors because of its rich redox reactions and good electrochemical activity. In addition, low toxicity, low cost of raw materials, high chemical stability, and simple synthesis approach make it a favorable electrode material for sensing [53,54,55,56,57]. Recently, Rabbani et al. prepared mesoporous NiCo_2_S_4_ nanoflakes through a facile hydrothermal approach and investigated their electrochemical behavior for cholesterol detection. The developed biosensor showed an excellent sensitivity of 8623.6 μAmM^−1^cm^−2^ in the wide linear range from 0.01 to 0.25 mM. The improved sensitivity has been considered due to the porous structure and large surface area [58]. The performance of the biosensor is presented in Figure 6.

**Figure 7:**
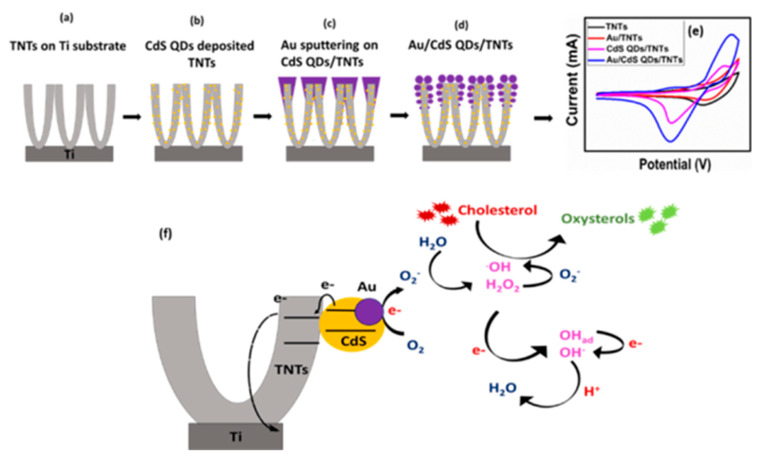
Schematic illustration of the (**a**–**d**) synthesis process of Au/CdS QDs/TNTs (**e**) CV response of different electrodes in the presence of cholesterol (**f**) oxidation reaction mechanism of cholesterol with the Au/CdS QDs/TNTs biosensor. Reprinted with permission from Ref. [59].

Khaliq et al. synthesized self-organized, highly ordered, and vertically aligned anodic titanium nanotube arrays decorated with gold sputtered cadmium dots (Au/CdS QDs/TNTs) for the investigation of non-enzymatic detection of cholesterol, as shown in Figure 7 [59]. The hybrid nanostructure’s (Au/CdS QDs/TNTs) electrode shows an increase in sensitivity for cholesterol (10,790 μAmM^−1^cm^−2^) in a wide linear range (0.024−1.2 mM) compared to the pristine sample. In addition, the electrode also demonstrates good reproducibility, thermal stability, and increased shelf life. The functionalized CdS QDs and Au NPs on TNTs provide fast electron transport and serve as passages for the redox species. The improved performance of the electrode can be considered due to the increase in active surface area, which results in inefficient detection of cholesterol. The schematic demonstrated the synthesis route of Au/CdS QDs/TNTs, CV response of different electrodes in the presence of cholesterol and oxidation reaction mechanism of cholesterol with the Au/CdS QDs/TNTs biosensor.

Cu_2_S, a well-known P-type semiconductor, is widely used in biosensors [60,61,62]. Its versatile morphology makes it very useful for electrochemical applications. Among various types of structures, the 3D structure made up of thickness nanoplates has good electrochemical properties. RongJi et al. synthesized Cu_2_S nanoroses made of thickness nanoplates on a Cu rod and used them for the non-enzymatic detection of cholesterol. The fabricated electrode Cu_2_S/CRIE demonstrated enhanced sensitivity of 62.5 µAmM^−1^ with a low limit of detection of about 0.1 µM. The electrode showed a sensitive and rapid response in a wide linear range from 0.01 to 6.8 mM, with good selectivity and stability [63]. Mir et al. reported an NIR emitting bovine serum albumin-functionalized Ag_2_S QDs for fast detection of cholesterol using fluorescence spectroscopy. The QDs having about 4.5 nm size exhibit an emission peak at 820 nm when excited at 420 nm. Under optimal conditions, the QDs show excellent sensitivity and selectivity for the detection of cholesterol up to the detection limit of 50 × 10^−9^ M [64].

### 2.3. Carbon Nanostructures Biosensor

Various carbon materials, including carbon nanotubes, carbon dots, graphene, graphene oxide, reduced graphene oxide, carbon nanofibers, and carbon black, have attracted much attention for the electrochemical sensing of a number of analytes, such as glucose, hydrogen peroxide, L-cysteine, cholesterol, etc. due to their large surface area, good thermal and chemical stability, and high electrical conductivity. For example, graphene has been used to immobilize biomolecules, such as DNA, antibodies, and enzymes, to develop efficient and selective biosensors. Bonanni et al. fabricated a graphene-based biosensor for the detection of DNA hybridization and polymorphism by employing electrochemical impedance spectroscopy. Mao, S. et al. fabricated a bio-field-effect transistor based on graphene decorated with gold nanoparticles for the detection of protein. Pankaj Gupta et al. developed a carbon nanotube decorated with Cu NPs-based microelectrodes for the rapid detection of glucose with excellent selectivity. Cheng-Shane Chu et al. fabricated a carbon quantum dots-based sensor for the detection of hydrogen peroxide. AL-Gahouari et al. fabricated a reduced graphene oxide–multiwall carbon nanotube-based sensor for the measurement of L-Cysteine and heavy metal ions [65,66,67,68,69,70,71].

Zhu et al. and Narvaez et al. reported various graphene and reduced graphene-based nanostructures with tuned electrochemical and photochemical properties for the detection of biomolecules. The charge transfer between the target molecule and graphene plays an important role in chemical sensing [72,73]. In addition, these materials can enhance biosensor performance due to their good electrochemical activity, wide potential window, and biocompatibility.

**Figure 8:**
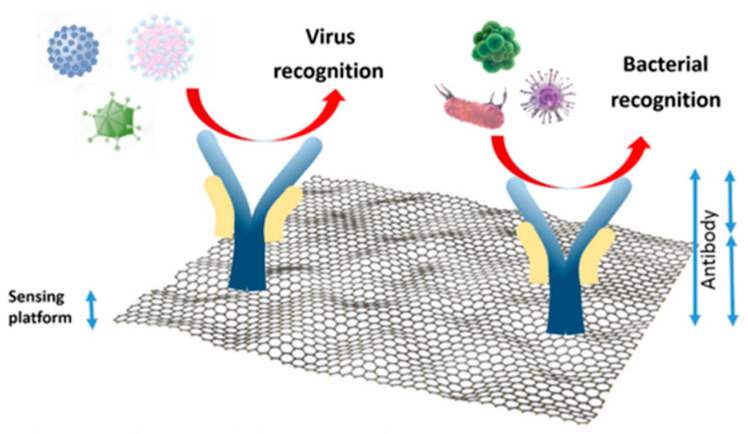
Schematic illustration of graphene-based biosensor. Reprinted with permission from Ref. [65].

Since the discovery of graphene, a number of articles on the design of efficient biosensors for the measurement of target biomolecules have been published, as shown in Figure 8. Graphene is one of the most important carbon materials due to its unique physical and chemical properties, including high charge mobility, zero-bandgap semiconductor, and high surface area. Chauhan et al. reviewed covalent and non-covalent functionalization of graphene with different organic molecules and metal/metal-oxide nanostructures for tuning their electrochemical properties for the detection of targeted biomolecules [74]. Janegitz et al. provided a comprehensive overview of the important properties of graphene for in vitro and in vivo electrochemical biosensing of antibodies, antigens, and DNA probes [75]. In order to tailor its electronic properties, doping of graphene is performed by nitrogen, which improves the interaction between the graphene surface and analytes. 

**Figure 9:**
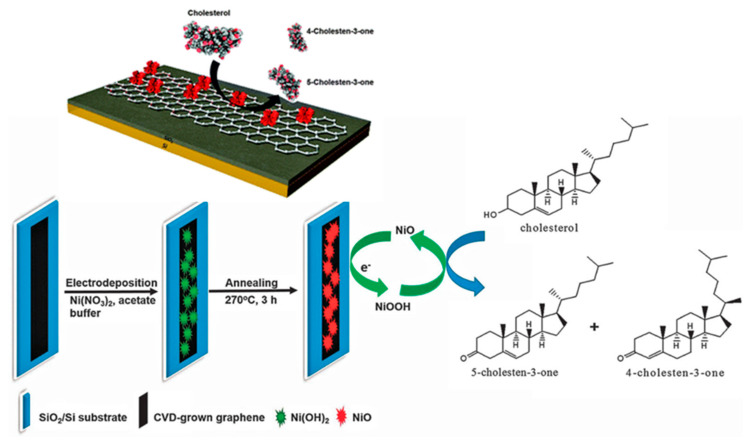
Schematic illustration of the fabrication of the NiO/graphene composite electrode for cholesterol sensing. Reprinted with permission from Ref. [76].

Rengaraj et al. reported a non-enzymatic electrochemical cholesterol sensor based on nickel oxide (NiO) functionalized on CVD-grown graphene (NiO/GR) nanocomposites, as demonstrated in Figure 9. The developed sensor demonstrated a high sensitivity of 40.6 mAmM^−1^cm^−2^ with a low detection limit of 0.13 mM due to the combined effects of NiO and graphene [76].

**Figure 10:**
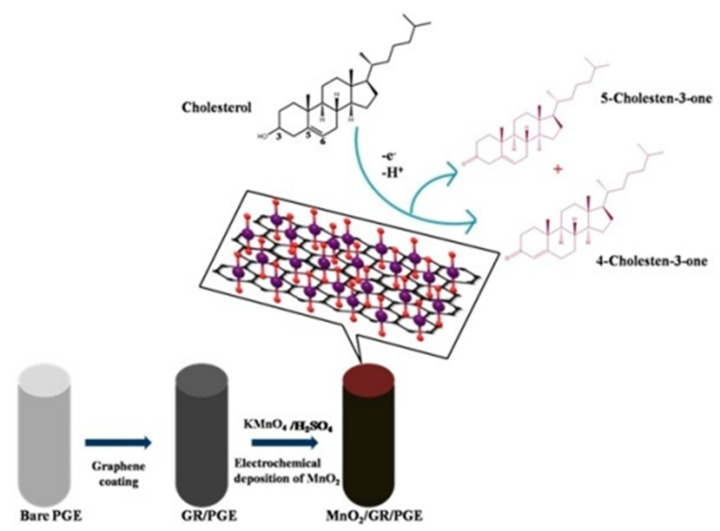
Schematic demonstration of the oxidation of cholesterol at MnO_2_/GR/PG. Reprinted with permission from Ref. [77].

Rison et al., as illustrated in Figure 10, reported a non-enzymatic cholesterol biosensor based on MnO_2_ nanoclusters immobilized on a graphene-modified pencil graphite electrode (MnO_2_/GR@PGE). This developed electrode was investigated for the detection of cholesterol. It has been found that the electrode showed a wide linear range for the measurement of cholesterol, from 12 × 10^−10^ M to 240 × 10^−10^ M, under optimum conditions, with a low detection limit of 0.42 nM. The results demonstrated that the electrode has excellent sensitivity for cholesterol [77].

**Figure 11:**
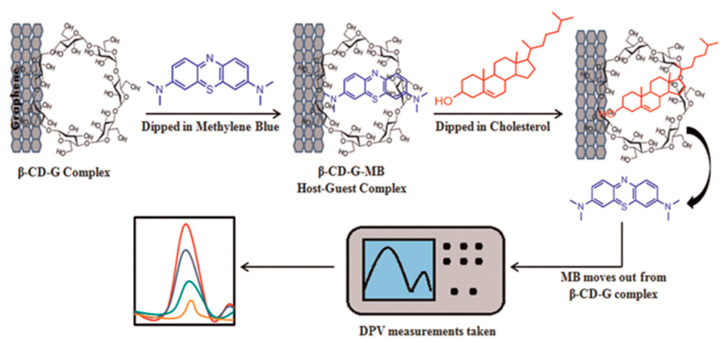
Demonstration of cholesterol sensing, using Grp-β-CD as the working electrode. Reprinted with permission from Ref. [78].

Agnihotri et al. presented a non-enzymatic approach toward cholesterol detectionusing β-cyclodextrin-functionalized graphene. This combination provided a platform for the electrochemical detection of cholesterol using methylene blue as a redox indicator, as displayed in Figure 11. The detection of cholesterol has been performed by using the differential pulse voltammetry technique [78].

**Figure 12:**
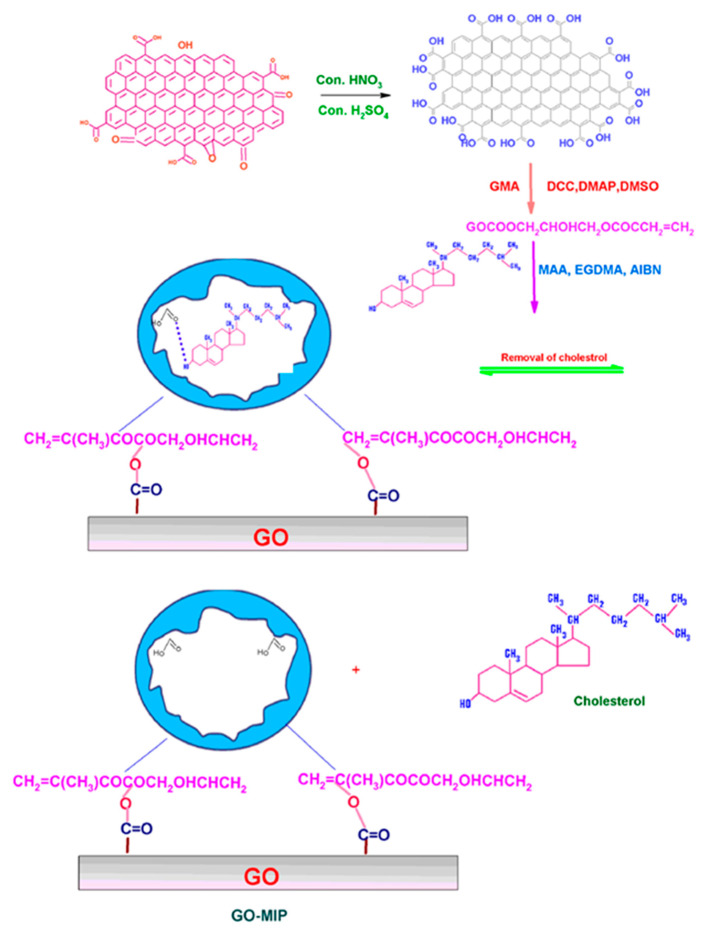
Schematic representation of the formation of GO-MIP. Reprinted with permission from Ref. [79].

Besides graphene, its derivatives graphene oxide (GO) and reduced graphene oxide (rGO) also have a lot of applications in the detection of different analytes. The oxygen groups on these materials are very suitable for improving electron transfer rates and water solubility, which ultimately enhances the sensitivity of the biosensor. Alexander et al. reported the fabrication of a cost-effective non-enzymatic cholesterol biosensor using graphene oxide-based molecular imprinted polymer (GO-MIP) as an active material, as illustrated in Figure 12. The constructed sensor exhibited good performance at pH 5.0, a rapid response time of ~2 min, and a low detection limit of 0.1 nM. Moreover, the electrode showed good selectivity and better performance in human blood samples, suggesting its practical pertinence [79].

It has already been reported that carbon nanotubes promote electron transfer rate, and therefore carbon nanotubes play a significant role in biosensing applications. Yang et al. synthesized a platinum nanoparticle-functionalized, layer-by-layer assembled CNT network and used it as a matrix to fabricate a non-enzymatic cholesterol biosensor. The 24-bilayer of CNT-based fabricated sensor showed a sensitivity of 8.7 lAmM^−1^cm^−2^, a wide linear range from 0.005 to 10 mM, and a very low limit of detection [80].

**Figure 13:**
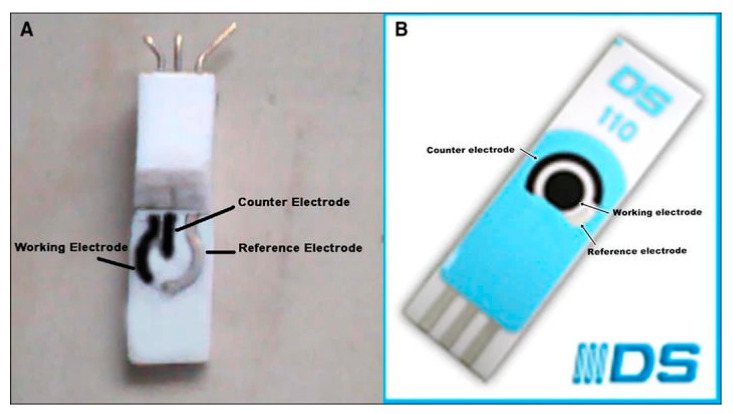
(**A**) Image of carbon nanotube (CCNT) modified electrode (**B**) Image of commercially available screen printed carbon nanotube electrode (DS110CNT). Reprinted with permission from Ref. [81].

Saha et al. fabricated a non-enzymatic cholesterol biosensor using carbon nanotubes obtained from coconut oil. The fabricated biosensor is shown in Figure 13. The modified electrode was tested by employing various electrochemical techniques such as cyclic voltammetry and differential pulse voltammetry in 0.001 M H_2_SO_4_ as electrolyte. The electrode showed a sensitivity of 15.31 ± 0.01 lAlM^−1^cm^−2^, a good response time of about 6 s, and a wide linear range from 1 to 50 µM [81]. Dey et al. also presented a Pt nanoparticle-functionalized graphene-based highly sensitive amperometric biosensor. The sensor displayed the best sensing response toward cholesterol.

**Figure 14:**
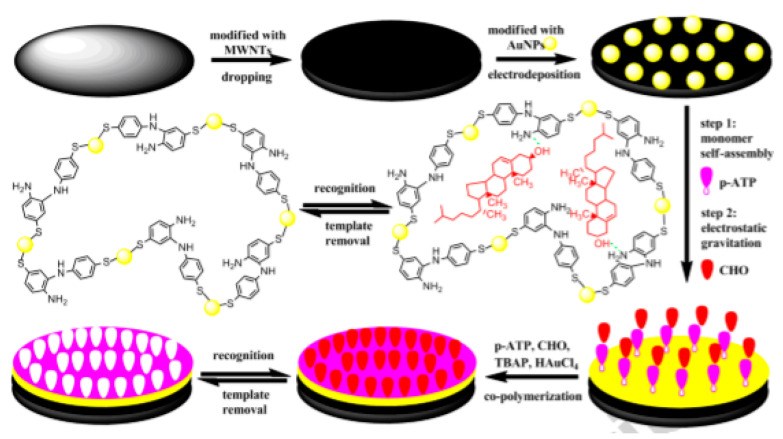
Schematic illustration of MIP/GCD/MWCNTs/AuNPs cholesterol biosensor. Reprinted with permission from Ref. [82].

The electrochemical properties of CNTs are strongly influenced by the basal and edge plane sites, and therefore they show poor response for electrochemical signals. Ji et al. functionalized multi-walled carbon nanotubes MWCNTs with gold nanoparticles (Au NPs) and improved the conductivity [82]. To fabricate a non-enzymatic cholesterol biosensor, a molecularly-imprinted polymer membrane on a glassy carbon electrode was modified with MWNTs/Au NPs, as shown in Figure 14. It has been found that the developed sensor exhibits good sensing response in a linear range between 1 × 10^−13^ and 1 × 10^−9^ mol L^−1^. The sensor exhibits excellent sensitivity, good stability, and selectivity.

**Figure 15:**
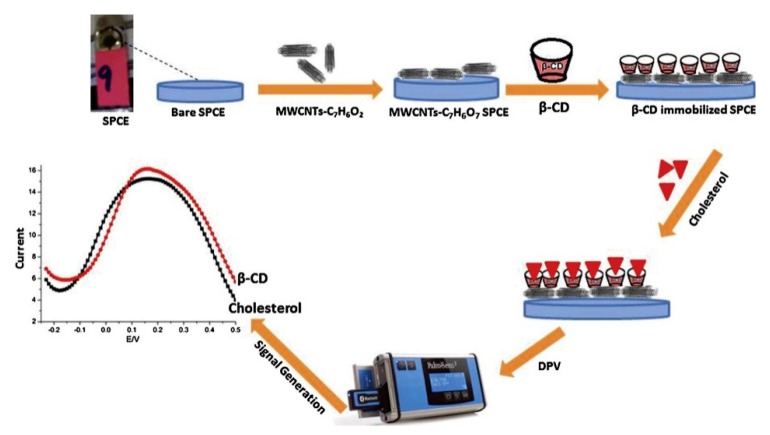
Schematic illustration of the preparation of SPCE/MWCNTs/β-cyclodextrin sensor for the measurement of cholesterol. Reprinted with permission from Ref. [83].

Nawaz et al. developed a MWCNTs- and β-cyclodextrin (β-CD)-based disposable screen printed carbon electrode (SPCE) for non-enzymatic sensing of cholesterol (Figure 15). β-CD was immobilized on benzoic acid-functionalized CNTs. The composite’s electrode can measure cholesterol from 1 nM to 3 μM with a lower detection limit of 0.5 nM. The electronic properties of CNTs and high affinity of β-CD toward cholesterol were utilized to produce an efficient and sensitive biosensor. The developed sensor is equally applicable for the determination of cholesterol in human serum, with a recovery of 94–96% and RDS of 4.5% [83].

### 2.4. Conducting Polymers and Cavity Molecules-Based Bio-Sensors

Nanostructured materials including conducting polymers (CPs), such as polyaniline (PANI), polypyrrole, etc., have become extremely essential in biosensor design. These materials have become very important for the development of different analyte-recognizing parts of biosensors and are synthesized very easily via electrochemical or chemical processes. The molecular chain structure of these materials can be modified by copolymerization or structural derivations [84,85,86,87]. The excellent mechanical properties of these materials make them very useful for the fabrication of sensors. Polymer-based nanocomposites due to their novel properties have motivated scientists to explore applications in various fields [88,89]. Among conducting polymers, PANI has high electrical conductivity as well as air stability and easy synthesis, and has become very significant as a result [90,91]. The copper oxide (CuO) nanoparticles being versatile material are gaining attention due to the demand for electronic devices. They have recently been used in several investigations on superconductors, catalysts, lithium-ion batteries, sensors, antibacterial agents and solar cells [92,93,94,95,96,97]. Hassine et al. synthesized CuO nanoparticles, PANI nanofibers, and murexide matrix for the development of a cholesterol biosensor. The prepared composite matrix was tested for the detection of cholesterol by using impedance spectroscopy technique. The fabricated sensor showed good performance with good stability and high sensitivity (5575 Ω/M) in a wide linear range from 0.5 nM to 50 mM [98].

**Figure 16:**
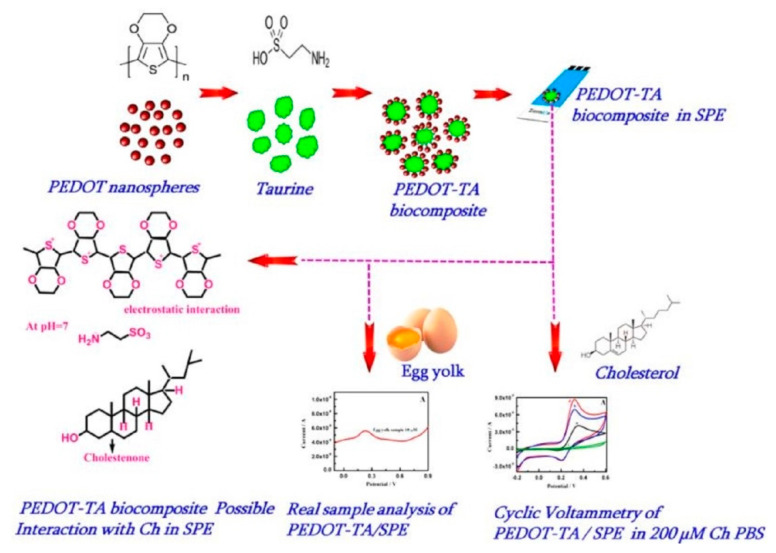
Schematic preparation of PEDOT-TA hybrid biocomposite selective sensing of cholesterol with possible interaction mechanism. Reprinted with permission from Ref. [99].

Thivya et al. prepared a poly(3,4-ethylenedioxythiophene) (PEDOT)-Taurine (TA) biocomposite coated screen printed electrode for non-enzymatic detection of cholesterol, shown in Figure 16. The electrostatic interactions between PEDOT and TA play the main role in the stability of the sensing matrix and efficient electrochemical measurement of cholesterol. The sensor is investigated using cyclic voltammetry, square wave voltammetry, and amperometry. The electrode shows a wider linear response and can be utilized for the measurement of cholesterol in egg yolk samples [99]. 

**Figure 17:**
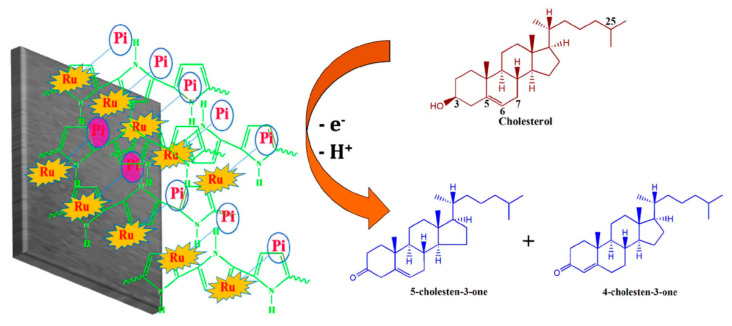
Schematic illustration for the probable redox mechanism of cholesterol on Ru-Pi-PPy/CFP electrode. Reprinted with permission from Ref. [100].

Akshaya et al. developed a cholesterol biosensor by electrodeposition of Ru-phosphate on polypyrrole (PPy)-modified carbon fiber paper (CFP) electrode [100]. Phosphate plays a vital role for fast and active non-enzymatic measurement of cholesterol (Figure 17). The developed Ru-Phosphate/PPy/CFP electrode was successfully used for the determination of cholesterol at an ultralow level in human blood serum. 

**Figure 18:**
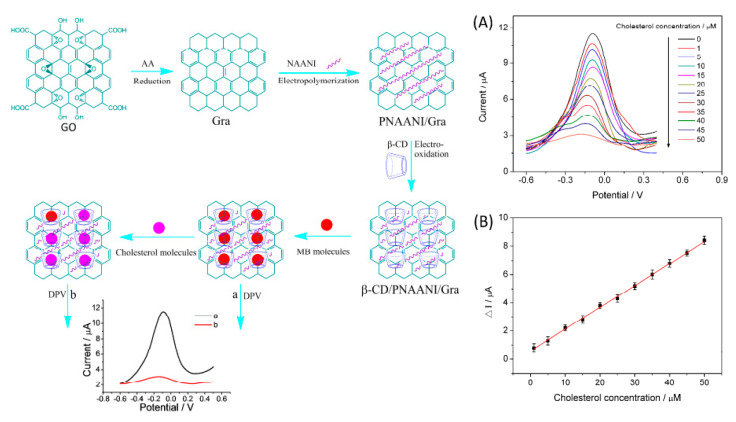
Schematic illustration of the preparation of β-cyclodextrin/poly(N-acetylaniline)/graphene-modified electrode and detection of cholesterol based on the competitive host–guest interaction between β-CD and MB (**left**). (**A**) DPV response with increasing concentration of cholesterol (**A**). Cholesterol measurement calibration curve (**B**). Reprinted with permission from Ref. [101].

Yang et al., using a competitive host–guest recognition mechanism, designed β-CD/poly(N-acetylaniline)/graphene-modified electrode for efficient and selective non-enzymatic detection of cholesterol, illustrated in Figure 18. The methylene blue (MB) molecule interacts with the hydrophobic cavity of β-CD, and the modified electrode shows a significant anodic current. The competitive interaction of the cholesterol with β-CD decreased the oxidation peak of MB. The developed electrode displays a linearity of 1.00 to 50.00 µM with a low detection limit of 0.50 µM and can be applied for cholesterol determination in serum samples [101].

**Figure 19:**
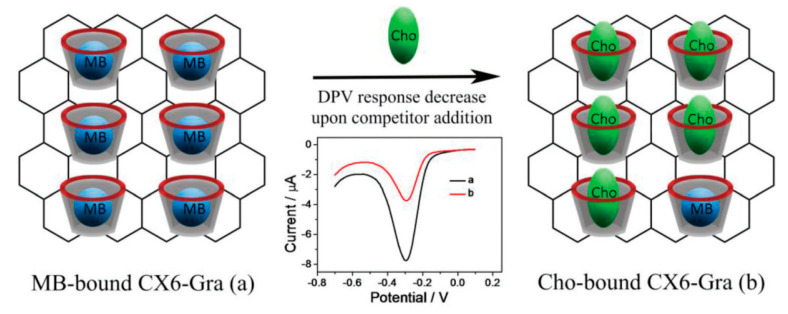
Schematic illustration of host–guest molecular detection of cholesterol (Cho) using CX6–Gra against MB. Reprinted with permission from Ref. [102].

Yang et al., in another effort, designed and developed a calix[6]arene-functionalized graphene-modified electrode that, following a competitive host–guest interaction mechanism, shows excellent sensitivity toward cholesterol. The cholesterol molecule displaces the MB molecule in the calix[6]arene–graphene complex, which leads to a “switch off” electrochemical response as displayed in Figure 19. The electrode shows a linear response from 0.50 to 50.00 μM with a low detection limit of 0.20 μM [102].

Ganganboina et al. covalently linked the βcyclodextrin (β-CD) with functionalized nitrogen-doped graphene quantum dots (N-GQD) with a mean particle size of 8.5 ± 0.5 nm. The electrode exhibits excellent sensitivity for the detection of cholesterol via a selective host–guest mechanism using a ferrocene (FC) redox indicator. The differential pulse voltammetry reveals a linear range from 0.5 to 100 μM of cholesterol concentration and a lower detection limit of 80 nM. The developed assay is highly selective against interfering species. The probe can be used for cholesterol measurement in spiked serum samples and has a good shelf life [103].

## 3. Strategies to Enhance the Performance of a Biosensor

The sensitivity of the biosensor- is a very important parameter to assess the performance of a biosensor. In this regard, researchers have considered various strategies to improve the sensitivity and reduce the interference from unwanted species in the biosensor. These include: (i) doping of metal ions in nanomaterials; (ii) annealing and exposing faces with different morphologies (1D, 2D, and 3D); (iii) introducing defects in nanomaterials; (iv) synthesized hybrid structures; and (v) controlling porous nanomaterials’ porosity. Various synthesis approaches such as electrodeposition, chemical vapor deposition, solvothermal, and hydrothermal methods have been adopted to synthesize these materials and utilized for the construction of biosensors. It has been reported that the mesoporous nanostructures increase the electrochemically active sites as well as surface area and play a significant role in the construction of non-enzymatic electrodes for cholesterol detection [104]. The defects in nanostructures lead to the delocalization of electron distribution and promotion of electron excitation responsible for fast charge transportation and increased conductivity [105]. The electrochemically active sites of the hybrid structures result in a high sensitivity, which makes them a potential candidate for the construction of cost-effective electrochemical biosensors [106]. To solve the biosensor stability problem, carbon nanomaterials, especially the oxygen-terminated functional groups of graphene, provide a suitable surface for modification with various functionalities. The tuning of their structural features provides a good platform for increasing the sensitivity and stability in non-enzymatic biosensors. Designing new cavity molecules for sensing of specific analytes utilizing a competitive host–guest mechanism is a highly promising approach to developing new highly selective and stable no-enzymatic bio-sensors.

## 4. Conclusions and Future Prospects

In this review article, we have provided a brief preview of non-enzymatic cholesterol biosensors reported in the literature, and described the current advances, challenges, and future prospects. Up till now, most of the presented cholesterol sensors have been enzymatic but their poor stability and high cost severely affect their performance and limit their use for practical applications. Therefore, researchers are increasingly working to fabricate reliable and robust non-enzymatic cholesterol biosensors by designing and developing novel nanomaterials. In the last two decades, various types of nanomaterials, such as metal, metal oxides, metal sulfides, carbon nanostructures, conducting polymers, and cavity molecules, have been developed and investigated for the detection of cholesterol. The recent progress on non-enzymatic biosensors reveals that the major challenge in non-enzymatic sensors is the selectivity for specific analytes and the ability to accurately measure the precise concentration of analytes. Although intensive research has been done for the development of selective electrode nanomaterials capable of cholesterol detection, some challenges still need to be resolved; firstly, there is a lack of studies on the toxicity of nanomaterials for the practical application in clinical laboratories; secondly, there is a need to understand the interactions between analytes and the host matrix, and more fundamental investigations need to be performed; thirdly, reproducibility is another factor that still requires more attention and research. Since nanomaterials can provide a suitable biocompatible environment, future research can be directed to designing in vivo biosensors for continuous and long-term monitoring of target analytes in real biological systems. Moreover, the development of novel nanomaterials capable of enzyme-like activity (mimics of cholesterol) will open new efficient pathways for the detection of cholesterol. In addition, functionalized nanomaterials having good conductivity and high surface area will provide a good platform for the construction of highly sensitive non-enzymatic cholesterol biosensors suitable for practical applications. A performance comparison is presented in Table 1.

## Figures and Tables

**Table 1:** Performance comparison of various non-enzymatic electrochemical biosensors for the determination of cholesterol.

Sr. No.	Electrode Material	Linear Range (µM)	Detection Limit (µM)	Applied Potential (V)	Sensitivity (µAmM^−1^cm^−2^)	Response Time (s)	References
1.	Ag NPs/polydopamine/graphenenanocomposites	0.005–997	0.68	0.0	140	>3	[15]
2.	Cu_2_O-TiO_2_	24.4–622	0.05	−0.46	6034.4	3	[35]
3.	Ag NPs-ZnO NRs	1–9	0.184	0.5	135.5	25	[41]
4.	PVIM-Co5POM-MNC	0.5–5000	1 × 10^−9^	—	64		[46]
5.	MB/BCD/Fe_3_O_4_/SPCE	2.88–150	2.88	−0.43	0.015	—	[47]
6.	NiCo_2_O_4_nanosheets	10–300	0.10	0.52	6150.7	<4	[58]
7.	NiCo_2_O_4_nanoflakes	10–250	0.01	0.45	8623.6	<2	[58]
8.	Au/CdS QDs/TNTs	0.024–1.2	0.012	—	10,790	~1	[59]
9.	Cu_2_S NRs/CRIE	10–6800	0.1	—	62.5	3	[63]
10.	NiO/CVD-grown Graphene	2–40	130	—	40,600	5	[76]
11.	MnO_2_/GR@PGE	0.0012	0.00042	—	—	—	[77]
12.	Graphene/β-CD	5–30	1	—	0.01	—	[78]
13.	GO-MIP	0.024	0.0001	—	—	~2	[79]
14.	CNT	1–50	0.01708	—	15.31	6	[80]
15.	Pt-CNT	5–10,000	2.8	0.7	8.7	—	[80]
16.	CuO/PANI/Mu	0.0005–50,000	—	—	5575 Ω/M	—	[81]
17.	β-CD/SPCE/CNTs	0.001–3	0.0005	—	—		[83]
18.	β-CD/N-GQDs	0.5–100	0.08	—	—		[103]
19.	Carbon containing composite	1–200	1.53	0.4	20000	-	[107]
20.	ZnO nanoparticles	0.001–0.5	—	0.355	23.7	>5	[108]
21.	Pencil Lead Electrode	625-9375	—	—	1422.22	—	[109]
22.	MoS_2_-Au	0.5–48	0.26	0.3	4460	—	[110]
23.	GCE	2–41	0.13	—	40.6	5	[111]
24.	Polyoxometalate/rGO composite	100–2 × 10^4^	1.02	0.02	95.6	5	[112]
25.	Cu/Ni bimetal-dispersed CNf/polymer nanocomposite	3.54–53,040	0.17	0.3	226.30	2.7	[113]
26.	PDMS/NiO/Pt	0.12–10.23	100	0.5	0.5	—	[114]
27.	Ag NPs/magnetic ferrous ferric oxide matrix	0.5–4 × 10^3^	0.2	−0.2	135	—	[115]
28.	CSPPy-g-C_3_N_4_H^+^/GCE	20–5000	8.0	—	645.7	~3	[116]
29.	Au-SPE/Pt/PDA	up to 500	10.5	—	14,300	<8	[117]
30.	NiO/ITO/glass	1000–12,000	500	—	63	5	[118]
31.	GCE/PTH	25–125	6.3	—	0.18	—	[119]
32.	G/PVP/PANI/paper	50–10,000	1	—	34.77	—	[120]
33.	Bi_2_O_2_CO_3_nanoplates	50–7400	10	—	139.5	~4	[121]
34.	G/Ti(G)-3DNS	50–8000	6	—	3.82	2	[122]
35.	PB/CPANI	600–6000	520	—	411.7	—	[123]
36.	(ZnO-CuO)/ITO/glass	500–12,000	—	—	760	5	[124]
37.	PBNPs	0–15,000	200	—	2.1	200	[125]
38.	ZnS_ZB_ nanotube	400–2000	440	—	598,000	<5	[126]
39.	GR-PtNPs hybrid	up to 12,000	0.2	—	2.07	—	[127]
40.	Pd-Pt NPs/GR nanocomposite	2.2–520	0.75	—	—	—	[128]
41.	Self-assembled GR	50–350	0.05	—	124.57	—	[129]
42.	GR/IL	0.25–215	500	—	4163	—	[130]
43.	CS/GR nanocomposites	5–1000	0.715	—	—	—	[131]
44.	ZnO/AgNWs/GR-CS nanocompostes	6.5–10,000	0.287	—	9.2	—	[132]
45.	GO-SH/AuNPs composite	50–11,450	0.0002	—	273	—	[133]
46.	NiOnanorods	640–10,300	650	—	120,000	—	[134]
47.	PANI/MWCNTs/Starch	32–5000	10	—	800	4–6	[135]
48.	ZnONanoporous Thin Film	1100–4830	—	0.9	3010	—	[136]
49.	Au/nPts	15–5000	15	—	226.2	—	[137]

## Data Availability

All the reported data available online.
